# Clinical Impact of EGFR Mutation Subtypes on Treatment Outcomes in Advanced Non-Small Cell Lung Cancer: An Austrian Real-World Study

**DOI:** 10.3390/cancers18020278

**Published:** 2026-01-16

**Authors:** Caroline Braschel, Hannah Fabikan, Vania Mikaela Rodriguez, Maximilian J. Hochmair, Oliver Illini, Leyla Ay, Christoph Weinlinger, Julie Krainer-Jacobs, Nino Müser, Arschang Valipour, Dagmar Krenbek

**Affiliations:** 1 Department of Pathology and Bacteriology, Klinik Floridsdorf, Vienna Healthcare Group, 1210 Vienna, Austria; dagmar.krenbek@gesundheitsverbund.at; 2 Karl Landsteiner Institute of Lung Research and Pulmonary Oncology, Klinik Floridsdorf, 1210 Vienna, Austria; hannah.fabikan@extern.gesundheitsverbund.at (H.F.); vaniamikaela.rodriguez@extern.gesundheitsverbund.at (V.M.R.); maximilian.hochmair@gesundheitsverbund.at (M.J.H.); oliver.illini@gesundheitsverbund.at (O.I.); leyla.ay@gesundheitsverbund.at (L.A.); christoph.weinlinger@extern.gesundheitsverbund.at (C.W.); julie.krainer@extern.gesundheitsverbund.at (J.K.-J.); nino.mueser@gesundheitsverbund.at (N.M.); arschang.valipour@gesundheitsverbund.at (A.V.); 3 Department of Respiratory and Critical Care Medicine, Klinik Floridsdorf, Vienna Healthcare Group, 1210 Vienna, Austria; 4 Karl Landsteiner Institute of Lung Research and Pulmonary Oncology, Klinik Ottakring, 1160 Vienna, Austria; 5 2nd Department of Internal Medicine with Pneumology, Klinik Ottakring, Vienna Healthcare Group, 1210 Vienna, Austria

**Keywords:** non-small-cell lung cancer, tyrosine-kinase inhibitors, targeted therapy, EGFR, exon 19 deletion, L858R point mutation, uncommon mutations

## Abstract

**Background**: Non-small cell lung cancer (NSCLC), particularly in advanced stages, has poor prognosis. The main objective of the study is to evaluate real-world treatment outcomes in advanced NSCLC patients harboring an EGFR mutation and being treated with TKIs. **Methods**: The EGFR mutation status was ascertained by next-generation sequencing. The observational cohort study used prospectively maintained registry data. Patient data were collected at two high-volume institutions in Austria between November 2020 and February 2025. The prevalence of EGFR mutations was 11% (145 out of 1267 patients). **Results**: Among 53 patients (stage IIIB or higher) with an EGFR mutation, median overall survival (OS) and median progression-free survival (PFS) were 17.7 months (95% CI: 10.4–24.9) and 14.2 months (95% CI: 7.4–20.9), respectively. A total of 36 patients harbored common EGFR mutations (exon 19 deletion or L858R point mutation) and exhibited a significantly better OS than those with an uncommon EGFR genotype (*p* < 0.005). Patients with exon 19 deletion (*n* = 25) showed the longest mOS, followed by those with L858R mutation (32.5 vs. 17 months). In multivariable analysis, the EGFR common mutation subtype (HR = 3.71 95%CI: 1.23–11.2) was associated with better OS. Patients with common EGFR genotypes, especially exon 19 deletion obtained longer OS and PFS compared with those with uncommon mutations in exon 18–21. **Conclusions**: The results underscore the prognostic role of distinct EGFR genotypes and the urgency of determining the mutation status in non-small cell lung cancer patients to ensure the best treatment decision. The study also highlights the challenges regarding to EGFR uncommon mutations and the resulting need for further research to investigate alternative treatment options.

## Simple Summary

The discovery of activating mutations in the epidermal growth factor receptor (EGFR) gene and development of targeted therapies such as EGFR tyrosine kinase inhibitors (TKIs) have had an enormous impact on treatment strategies for advanced non-small cell lung cancer (NSCLC) patients. TKIs are designed to block specific mutations and prevent cancer from growing. In particular, common mutations (exon 19 deletion and L858R point mutation) are strong predictors for favorable survival outcome due to positive treatment response. The present study evaluated real-world survival outcomes of patients with advanced EGFR-mutated NSCLC treated with EGFR TKIs in Austria, with a specific focus on comparing outcomes between patients with common and rare EGFR mutation subtypes outside of clinical trial settings.

## 1. Introduction

Lung cancer is, with 1.8 million deaths, the leading cause of cancer-related deaths [1]. As the data indicate, lung carcinoma is one of the top aggressive cancers with poor outcomes. Almost half of non-small cell lung cancer (NSCLC) patients have stage IV at initial diagnosis [2]. The 5-year survival rate persists at a very low level [3], and roughly 75% of patients diagnosed with advanced lung cancer have a bad prognosis [4]. Chemotherapy only achieves median survival rates up to 14 months [5]. The approval of epidermal growth factor receptor (EGFR)-tyrosine kinase inhibitors (TKIs) led to a decrease in mortality and transformed the landscape of NSCLC treatment and prognosis [6,7]. According to phase III clinical trials, EGFR-TKIs are superior to chemotherapy regarding to prolonging progression-free survival (PFS) [8,9] and overall survival (OS) [10].

EGFR mutations are the second most prevalent oncogenic drivers in NSCLC. The most common activating mutations (exon 19 deletion and L858R point mutation) comprise the vast majority of EGFR mutations (80–85%), whereas uncommon mutations (Exon 18, 20, 19, and 21) are less frequent (15–20%) [11,12]. EGFR exon 20 insertions are the third most common genotype and occur in 1–10% of NSCLC patients [13]. Since most of the patients with EGFR-positive NSCLC carry either an exon 19 deletion or exon 21 L858R point mutation, data on uncommon EGFR genotypes are lacking [14].

Common mutations are defined as predictors for good clinical response to EGFR-TKIs [15]. As reported by previous studies, NSCLC patients with exon 19 deletion showed better OS and PFS than patients with L858R mutation [16,17]. Based on a meta-analysis, the EGFR mutation prevalence is higher in the Asian population (49.1%) than in Western countries (12.8%) [7]. Therefore, data on survival outcome for patients in Europe harboring advanced NSCLC and treated with EGFR-TKIs in first-line palliative treatment have been lacking.

It is expected that the results of this prospective real-world study will provide more evidence for the use of EGFR-TKIs in first-line treatment in European advanced NSCLC patients with EGFR mutations. Additionally, the comparison of the EGFR genotypes regarding survival outcome will deliver important prognostic information.

## 2. Materials and Methods

This observational cohort study evaluated real-world overall survival (rwOS) and real-world progression-free survival (rwPFS) survival outcome of EGFR-positive advanced (stage IIIB or higher) NSCLC patients who were treated with TKIs as the first line, stratified by EGFR mutation subtype. The study was conducted with a registry framework, and all patients signed an Informed Consent Form (ICF). Patient data were collected at two high-volume clinics in Austria (Department of Respiratory and Critical Care Medicine, Klinik Floridsdorf, Vienna; Department of Internal Medicine with Pulmonology, Klinik Ottakring, Vienna) between November 2020 and the last follow-up in February 2025. The EGFR mutation status was ascertained by next-generation sequencing from tumor tissue (ThermoFisher OncomineTM Focus assay, Ion AmpliSeqTM lung cancer panel, Waltham, MA USA). Clinical characteristics and treatment data were predefined and retrospectively extracted from Longitudinal Analysis of LUng CAncer registry data (LALUCA, EK 20-061-VK, city of Vienna, Austria) [18], which was implemented by the Karl Landsteiner Institute for Lung Research and Pulmonary Oncology in Vienna, Austria.

Survival outcomes were estimated using Kaplan–Meier methods for rwOS and rwPFS. Survival analyses compared patients with EGFR exon 19 deletions, EGFR exon 21 L858R mutations, and uncommon EGFR mutations located in exons 18–21. Univariate and multivariable Cox proportional hazards regression was calculated to identify potential prognostic factors associated with OS and PFS. Hazard ratios with 95% confidence intervals were visualized using forest plots.

### Statistical Analysis

Statistical analysis was performed with the open-source software R-4.4.1 and IBM SPSS Statistics version 30.0. Demographic and clinical characteristics of the study population (*n* = 53) were summarized descriptively. Group comparisons between patients with common and uncommon EGFR mutations were performed using Fisher’s exact test, as appropriate.

The influence of TKIs as well as EGFR mutation subtypes on OS and PFS in EGFR-positive NSCLC patients was estimated with Kaplan–Meier analyses. Potential differences between survival curves were assessed using the log-rank test. OS was calculated from the start of first-line EGFR-TKI treatment to death from any cause. Participants who were alive at the time of analysis were censored at the last date of last known follow-up. PFS was defined as the time from the initiation of first-line therapy to documented disease progression or death from any cause. Disease progression was defined as a change or discontinuation of first-line therapy with progression explicitly documented as the reason for treatment change in the medical records. Death was considered as a progression event. Patients who discontinued or changed therapy for reasons other than documented disease progression, as well as patients remaining on first-line therapy at the time of data cut-off, were censored at the date of treatment change or last follow-up, respectively. The Cox proportional hazard regression model was assessed for the identification of potential prognostic factors associated with OS and PFS. For *p*-values, the established 5% α-level was used in order to draw conclusions about statistical significance.

## 3. Results

In total, 145 patients with an EGFR mutation (11%) were identified out of 1267 patients (844 adenocarcinoma, 302 squamous cell carcinoma, 117 NOS or large cell carcinoma, 4 adenosquamous carcinoma) with NSCLC. The diagnosed EGFR variants were categorized into EGFR common und EGFR uncommon mutations, shown in Table 1. Of the 145 patients with an EGFR mutation, 87 (60%) had a common EGFR mutation and 58 (40%) harbored an uncommon EGFR mutation.

**Figure 1:**
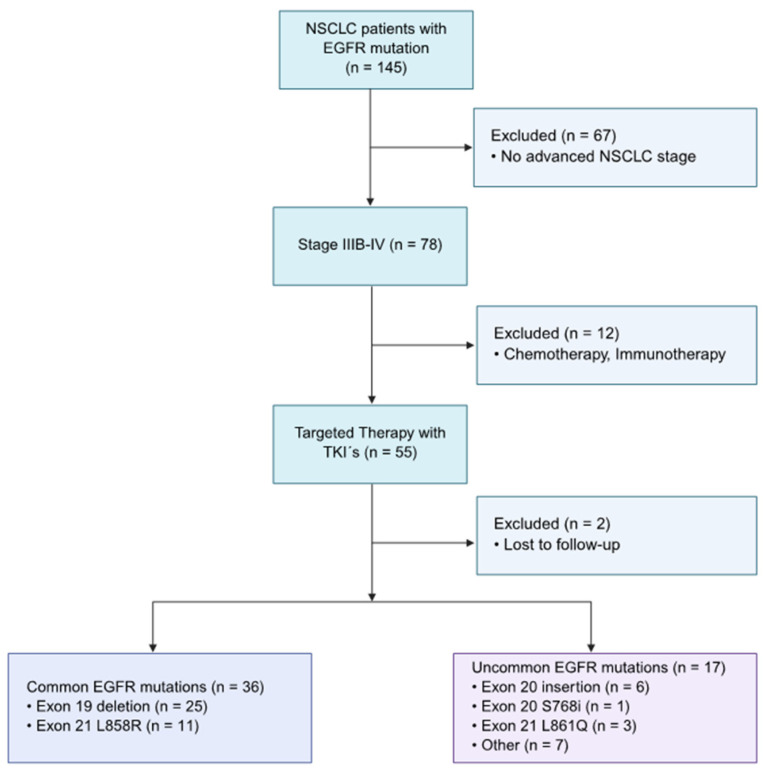
Flow chart of the study population, based on inclusion and exclusion criteria. Inclusion criteria: NSCLC at an advanced stage (IIIB or higher), TKI therapy as first line with known start and end date, and harboring an EGFR mutation. The eligible patients (*n* = 53) included in the study were stratified into the two categories: common (*n* = 36) and uncommon EGFR mutations (*n* = 17).

Among 145 patients with pathologically confirmed advanced NSCLC with known EGFR mutation status, 53 cases were included for evaluation, based on the following eligibility criteria: harboring advanced NSCLC, somatic EGFR mutation, and having received TKIs as first-line treatment (palliative therapy), illustrated in Figure 1. The final sample size was subdivided into the common mutation (*n* = 36) and uncommon mutation (*n* = 17) groups.

Demographic and clinical characteristics are detailed in Table 2; tumor characteristics, including the stage at initial diagnosis, location of metastasis, sites of metastasis, histologic subtype and PD-L1 status are presented in Table 3. The patients received second-generation and third-generation TKIs as the first line, which are summarized in Table 4, including response.

The median age at initial diagnosis was 73 years (range, 52–85 years); patients ≥65 years (66%) were predominant. The vast majority of the patients were Caucasian (91%) and female (60%). Approximately half of the patients (57%) had a body mass index (BMI) ranging from 18.5 to 24.9. Most of the patients had never smoked (43%) or were former smokers (38%); 19% were heavy smokers (≥30 pack years). Based on performance status (ECOG), the majority of the patients had ECOGs of 0 (60%), 30% had ECOGs of 1, 6% had ECOGs ≥ 2, and 4% were not evaluated. Most of the patients with exon 19 deletion were female (68%), whereas the gender dissemination of patients with an exon 21 L858R mutation or uncommon mutations was almost equally distributed.

All variables (age, sex, ethnicity, smoking status, pack years, BMI, and ECOG performance status) did not significantly differ between the EGFR exon 19 deletion, EGFR exon 21 L858R mutation, and uncommon mutation (*p* > 0.05) groups.

As shown in Table 3, most of the patients (93%) had stage IVA (42%) or stage IVB (51%); only 8% had stage IIIB. Most patients harbored an adenocarcinoma (91%), followed by squamous cell carcinoma (6%) and large cell carcinoma (4%). A negative PDL-1 expression (<1%) was found in 25 patients (47%); 21 patients (40%) had a PDL-1 expression of 1–49%. In total, 6% had a high (50–89%) or a very high PDL-1 expression (≥90%). The primary sites of metastasis were the pleura (42%), brain (26%), and bones (34%). Rare locations of metastasis were distant lymph nodes (9%), the adrenal gland (6%), and the liver (11%). The cumulative percentages for metastatic locations will not sum to 100%, since patients can exhibit multiple metastatic sites simultaneously.

All variables (stage at initial diagnosis, histologic type, PDL-1 status, location, and site of metastasis) did not significantly differ between the EGFR exon 19 deletion, EGFR exon 21 L858R mutation, and uncommon mutation (*p* > 0.05) groups.

Patients included in this study received second-generation and third-generation TKIs as the first line. Details regarding the treatment pattern are summarized in Table 4. Most of the study population received Osimertinib (third-generation TKI) (58%) or Afatinib (second-generation TKI) (32%). Of the 53 patients, 4 (8%) out of 6 patients with an exon 20 insertion were treated with Mobocertinib. One patient additionally harbored an ALK translocation and was treated with Brigatinib, a second-generation ALK TKI. Notably, first-line EGFR TKI selection varied according to EGFR mutation status, with more frequent use of Osimertinib in patients with common mutations and preferential use of Mobocertinib in patients with uncommon mutations.

None of the patients had a complete response at the first line; 36% had a partial response, followed by 11% with stable disease and 4% with progressive disease. A substantial proportion of patients (49%) had unknown best response due to incomplete documentation in routine clinical practice.

### 3.1. Survival Analysis

#### 3.1.1. Overall Survival Analysis

**Figure 2:**
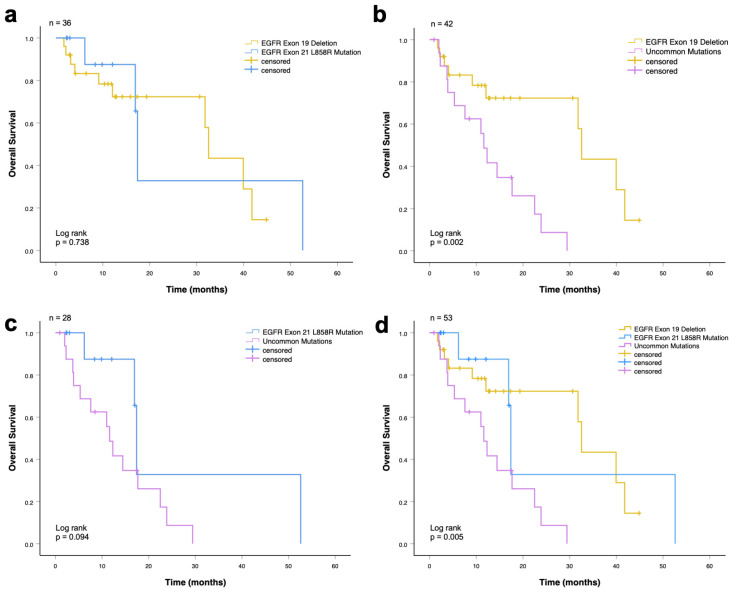
Overall survival among advanced NSCLC patients who received EGFR-TKI (first-line) according to EGFR mutations. (**a**) Patients with exon 19 deletion and exon 21 L858R mutation (*p* = 0.74). (**b**) Patients with exon 19 deletion and uncommon mutations (*p* = 0.002). (**c**) Patients with exon 21 L858R mutation and uncommon mutations (*p* = 0.094). (**d**) Patients with common (exon 19 deletion and exon 21 L858R mutation) and uncommon mutations (*p* = 0.005).

The median rwOS for the whole cohort (*n* = 53) was 17.7 months (95% CI, 10.4–24.9 months). Patients harboring an EGFR exon 19 deletion had a significantly longer overall survival compared with patients with EGFR uncommon mutations (32.5 vs. 11.6 months, *p* = 0.002; Figure 2b). There was no significant difference in the median overall survival between patients with EGFR exon 21 L858R mutation and uncommon mutations (17.4 vs. 11.6 months, *p* = 0.094; Figure 2c), and between the EGFR exon 19 deletion and EGFR exon 21 L858R mutation groups (32.5 vs. 17.4 months, *p* = 0.74; Figure 2a). In the analysis including all three mutation groups (exon 19 deletion, exon 21 L858R, and uncommon EGFR mutations), a statistically significant difference in median overall survival was observed (32.5 vs. 17.4 vs. 11.6 months; *p* = 0.005; Figure 2d). Patients with common EGFR mutations demonstrated superior OS compared with those with uncommon EGFR mutations, while survival outcomes between exon 19 deletion and exon 21 L858R mutations were comparable.

A summary of the median OS times is shown in Table 5, including the respective 95% CI. EGFR exon 19 deletion showed the longest median overall survival with 32.5 months (CI: 30.8–34.3 months), followed by EGFR exon 21 L858R mutation with 17.4 months (CI: 17.0–18.1 months). The shortest overall survival was in patients with uncommon mutations, with 11.6 months (CI: 9.2–14.0 months).

The univariate and multivariable Cox proportional hazard analysis of overall survival, including the variables age, sex, histologic type, BMI, ECOG performance status, sites of metastasis, PDL-1 expression, and EGFR mutation subtype, is shown in Table 6. The univariate analysis revealed that overall survival was longer in patients with a common mutation (HR: 3.7; 95% CI: 1.6–8.7; *p* = 0.002), those with an ECOG status of 0 (HR: 2.3; 95% CI: 1.1–4.5; *p* = 0.018), and patients with adenocarcinoma (HR: 4.3; 95% CI: 1.4–14; *p* = 0.013). In accordance with the univariate analysis, the multivariable analysis showed that NSCLC patients with a common mutation had a longer overall survival compared to the uncommon mutation subgroup (HR: 3.7; 95% CI: 1.23–11.2; *p* = 0.02). No significant difference regarding OS could be identified for age, sex, histologic type, ECOG status, sites of metastasis, PDL-1 expression, or BMI.

**Figure 3:**
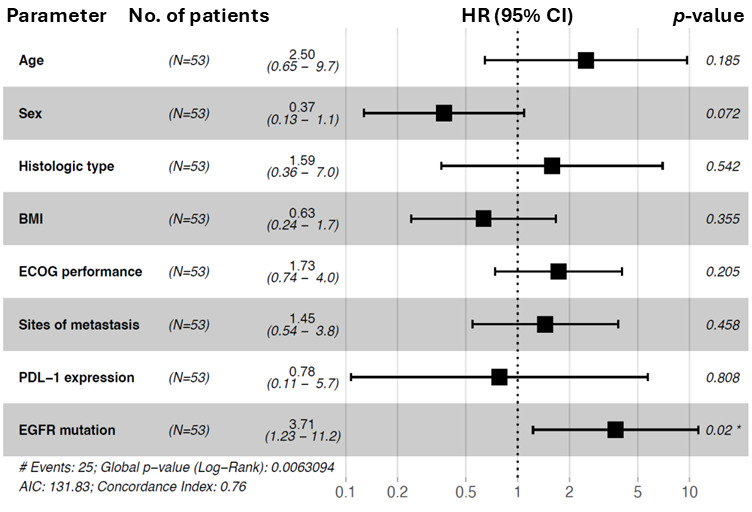
Forest plot of hazard ratios for overall survival (OS) for subgroup analysis (*n* = 53). The forest plot was used to show the prognostic association in different subgroups for overall survival. Patient numbers (*n* = 53), hazard ratios (HRs), *p* values, and 95% CIs (confidence intervals) are shown. Subgroups: age (reference: <60), sex (reference: male), histologic type (reference: ADC), BMI (reference: <25), ECOG status (reference: 0), sites of metastasis (reference: ≤1), PDL-1 expression (reference: 0–49%), and EGFR mutation (reference: common). Forest plot of hazard ratios from univariable Cox regression analysis. * indicates statistical significance at *p* < 0.05. # indicates the total number of events observed in the cohort.

In conclusion, an EGFR common mutation subtype (HR = 3.71) was an independent predictor of better overall survival. The results are visualized in Figure 3.

#### 3.1.2. Progression-Free Survival Analysis

**Figure 4:**
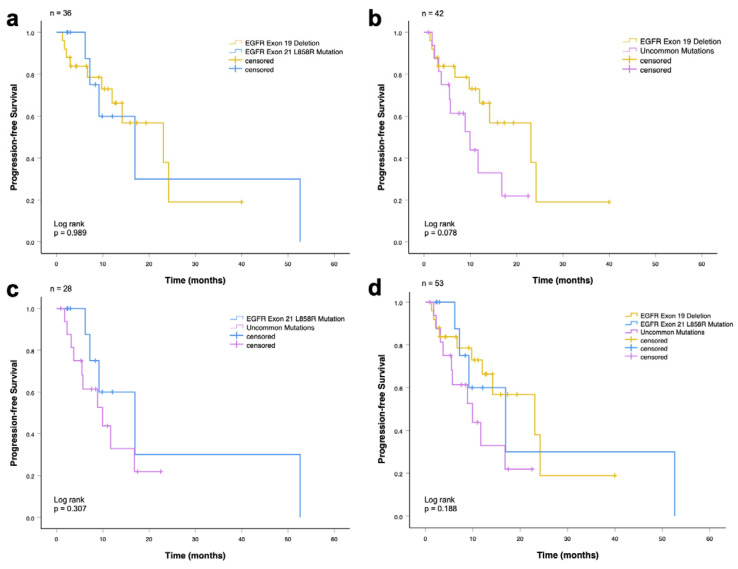
Progression-free survival among advanced NSCLC patients who received EGFR-TKI (first-line) according to EGFR mutations. (**a**) Patients with exon 19 deletion and exon 21 L858R mutation (*p* = 0.989). (**b**) Patients with exon 19 deletion and uncommon mutations (*p* = 0.078). (**c**) Patients with exon 21 L858R mutation and uncommon mutations (*p* = 0.307). (**d**) Patients with common (exon 19 deletion and exon 21 L858R mutation) and uncommon mutations (*p* = 0.188).

The median rwPFS in the entire cohort (*n* = 53) was 14.2 months (95% CI: 7.4–20.9 months). There was no significant difference in the median progression-free survival between all groups: patients with EGFR exon 19 deletion and EGFR exon 21 L858R mutation (23.1 vs. 17.0 months, *p* = 0.989; Figure 4a), patients with EGFR exon 19 deletion and EGFR uncommon mutations (23.1 vs. 10.0 months, *p* = 0.078; Figure 4b), and patients with EGFR exon 21 L858R mutation and uncommon mutations (17.0 vs. 10.0 months, *p* = 0.307; Figure 4c).

In the analysis including all three EGFR mutation subgroups (exon 19 deletion, exon 21 L858R, and uncommon mutations), no statistically significant difference in progression-free survival was observed (23.1 vs. 17.0 vs. 10.0 months; *p* = 0.188; Figure 4d).

Median PFS times are shown in Table 7, including the respective 95% CI. EGFR exon 19 deletion showed the longest median progression-free survival with 23.1 months (CI: 6.8–39.4 months), followed by EGFR exon 21 L858R mutation with 17.0 months (CI: 5.2–28.7 months). The shortest OS (10 months) also had patients with uncommon mutations (CI: 3.4–16.4 months).

The univariate and multivariable Cox proportional hazard analysis of progression-free survival, including the variables of age, sex, histologic type, BMI, ECOG performance status, sites of metastasis, PDL-1 expression, and EGFR mutation subtype, is demonstrated in Table 8. Both univariate and multivariable analysis revealed no significant associations.

**Figure 5:**
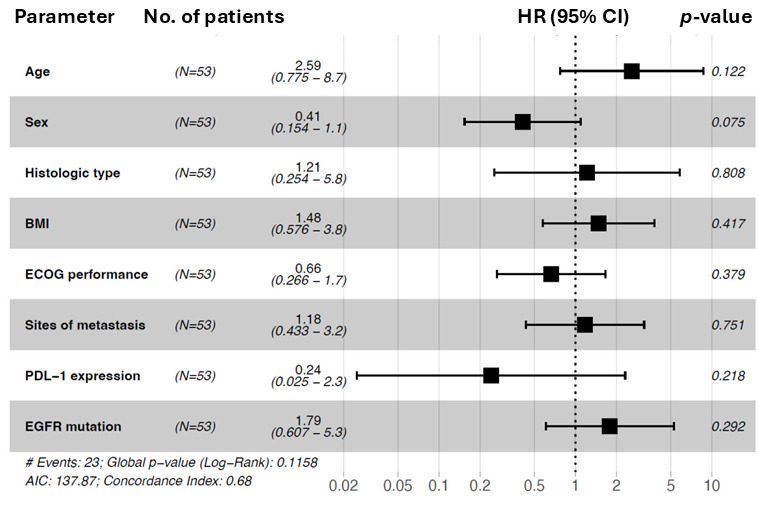
Forest plot of hazard ratios for progression-free survival (PFS) for subgroup analysis (*n* = 53). The forest plot was used to demonstrate the prognostic association in different subgroups for progression-free survival. Patient numbers (*n* = 53), hazard ratios (HRs), *p* values, and 95% confidence intervals are shown. Subgroups: age (Reference: <60), sex (Reference: male), histologic type (reference: ADC), BMI (reference: <25), ECOG status (reference: 0), sites of metastasis (reference: ≤1), PDL-1 expression (reference: 0–49%), and EGFR mutation (reference: common). Forest plot of hazard ratios from univariable Cox regression analysis. # indicates the total number of events observed in the cohort.

The results are visualized in Figure 5.

## 4. Discussion

In the present study, we examined a prospective cohort of 53 patients with advanced NSCLC harboring an EGFR exon 18–21 mutation from two high-volume institutions in Vienna, Austria.

The beneficial survival outcomes of EGFR-TKIs in NSCLC patients at the first line, as well as the prognostic impact of clinicopathological characteristics and EGFR subtype differences, have been examined previously but predominantly in Asian, Middle East and North Africa, Moroccan, and especially Chinese populations [19,20,21]. To the best of our knowledge, this is one of the first real-world analyses to investigate the survival outcome after first-line EGFR-TKI treatment (second- and third-generation) as well as the prognostic role of EGFR genotypes and clinical and pathological characteristics in advanced NSCLC patients in Austria.

In the present cohort, the majority of the patients were female (60%), Caucasians (91%), and never or former smokers (81%). Differences between common and uncommon EGFR mutations failed to reach significance between age groups, sexes, ethnicities, smokers and non-smokers, pack years, or for varying ECOG status and BMI. Previous studies unveiled an association between distinct EGFR genotypes and smoking habits. Albeit not nominally significant, more than half of the patients with EGFR uncommon mutations are smokers or former smokers in contrast to patients harboring common mutations, where more than half of the patients are never smokers, supporting prior evidence [15,19,22,23,24,25]. The higher number of females in our sample reflects the previously shown [19,22,25,26,27] increased likelihood in women with NSCLC of developing the EGFR mutation compared to men. Also, in line with previous findings, we provide tentative evidence for higher rates of EGFR exon 19 deletion within females [19,22,28]. A substantial number of the patients had adenocarcinoma (91%), stage IV (93%), and one metastasis (55%). For both the clinicopathological characteristics and tumor characteristics, no significant differences in the number of common and uncommon mutations could be found.

Within the scope of the present study, we also investigated potential prognostic factors. Multivariable Cox regressions revealed that an EGFR common genotype is an independent prognostic factor for improved median overall survival (mOS). No significant association could be found concerning age, sex, histologic type, ECOG performance status, BMI, sites of metastasis, and PDL-1 expression. The observations align with a previous study, where female gender was additionally mentioned as an independent prognostic factor for improved overall survival [19]. The strongest independent factor for prolonged overall survival is the EGFR common genotype, which is consistent with previous investigations [2,29]. Regarding median progression-free survival (mPFS), independent prognostic factors could not be identified.

The survival analysis of the study demonstrated that patients with common EGFR mutations exhibited significantly longer mOS compared with those with an uncommon EGFR genotype. Moreover, within EGFR common mutations, exon 19 deletion surpassed (although not nominally significant) the mOS compared to the exon 21 L858R mutation cohort. These results are concordant with previous real-world studies and clinical trials [4,19,20,29,30]. Patients with EGFR exon 19 deletion achieved better mPFS, followed by EGFR exon 21 L858R; uncommon mutations had the poorest mPFS. These findings are overall in line with foregoing studies [16,17,30] but should be interpreted with caution due to the biological heterogeneity of uncommon EGFR mutations. Based on available evidence, selected uncommon EGFR mutations have demonstrated sensitivity to EGFR TKIs. In particular, retrospective data suggest clinical activity of Osimertinib in certain uncommon EGFR mutations, as reported in the UNICORN study [31], while Afatinib has shown efficacy in patients with uncommon and compound EGFR mutations in pooled analyses of the LUX-Lung 2, 3, and 6 trials [32]. Accordingly, EGFR TKIs were used in selected patients harboring rare EGFR mutations in the present study.

Therapeutic approaches for EGFR-positive NSCLC continue to evolve. Recent studies such as FLAURA2 [33] and MARIPOSA [34] suggest improved outcomes with treatment combination in patients with common EGFR mutations, whereas treatment options for uncommon EGFR mutations remain highly dependent on mutation subtypes and need further investigation.

### Limitations

Some limitations have to be acknowledged. First, the relatively small sample size may restrict the statistical power of several subgroup analyses. In addition, treatment options have changed over the study period. The vast majority of patients were treated with Afatinib (second-generation) or Osimertinib (third-generation), which may have influenced survival outcomes. According to previous study data, the treatment with Osimertinib as the first line resulted in a significantly longer mPFS and mOS than with comparator TKIs [10,35,36]. In addition, the observed OS and PFS outcomes may reflect treatment-related factors rather than isolated biological effects of EGFR mutation subtype. Moreover, therapy options for patients harboring rare EGFR mutations remain more restricted than those for common mutations, which may be one reason for poorer survival outcomes. In addition, patients carrying rare EGFR mutations are less responsive to TKIs [22,24]. In the present analysis, uncommon EGFR mutations were analyzed as a pooled group, which may have introduced heterogeneity and influenced effect estimates, particularly if outcomes were driven by subtypes with lower sensitivity to TKIs.

## 5. Conclusions

The findings of the present survival analysis in a real-world setting are in agreement with the results of clinical trials. Our results underline the prognostic role of distinct EGFR genotypes and the relevance of corresponding analyses in NSCLC patients. The study also highlights the challenges regarding to EGFR uncommon mutations and the resulting need for further research to investigate alternative treatment options.

## Figures and Tables

**Table 1:** Occurrence of different EGFR mutations in the study population. Mutation types are subdivided in EGFR common mutations and uncommon mutations.

EGFR Mutation	All Patients (*n* = 145)
**Common**, *n* (%)	
Exon 19 deletion	58 (40)
Exon 21 L858R mutation	29 (20)
**Uncommon**, *n* (%)	
Exon 20 insertion	28 (19)
Exon 21 L861Q mutation	6 (4)
Exon 20 S768i mutation	2 (1)
Other ^1^	23 (16)

^1^ Other: rare mutations in exon 18–21.

**Table 2:** Demographic and clinical characteristics of the study population (*n* = 53).

Demographic and Clinical Characteristics	No. of Patients	Exon 19 Deletion	L858R Mutation	Uncommon Mutations	*p* Value ^1^
**Total**	53	25 (47%)	11 (21%)	17 (32%)	
**Age, years**					
Median (range)	73 (52–85)	66 (52–82)	74 (59–85)	73 (57–83)	
**Age group,** ***n*** **(%)**					0.1584
<65	18 (34)	12 (48)	2 (18)	4 (24)	
≥65	35 (66)	13 (52)	9 (82)	13 (76)	
**Sex,** ***n*** **(%)**					0.5824
Male	21 (40)	8 (32)	5 (45)	8 (47)	
Female	32 (60)	17 (68)	6 (55)	9 (53)	
**Ethnicity,** ***n*** **(%)**					0.3823
Asian	2 (4)	1 (4)	-	1 (6)	
Caucasian	48 (91)	24 (96)	10 (91)	14 (82)	
Unknown	3 (5)	-	1 (9)	2 (12)	
**Smoking status,** ***n*** **(%)**					0.2252
Never a smoker	23 (43)	13 (52)	6 (55)	4 (24)	
Former smoker	20 (38)	7 (28)	3 (27)	10 (59)	
Smoker	9 (17)	5 (20)	2 (18)	2 (12)	
Unknown	1 (2)	-	-	1 (6)	
**Pack years,** ***n*** **(%)**					0.4331
Light smoker (1–29 py)	18 (34)	8 (32)	2 (18)	8 (47)	
Heavy smoker (≥30 py)	10 (19)	4 (16)	2 (18)	4 (24)	
Unknown	25 (47)	13 (52)	7 (64)	5 (29)	
**ECOG status at diagnosis,** ***n*** **(%)**					0.6057
0	32 (60)	17 (68)	6 (55)	9 (53)	
1	16 (30)	5 (20)	4 (36)	7 (41)	
≥2	3 (6)	1 (4)	1 (9)	1 (6)	
Unknown	2 (4)	2 (8)	-	-	
**BMI,** ***n*** **(%)**					0.2433
Underweight (<18.5)	1 (2)	1 (4)	-	-	
Normal range (18.5–24.9)	30 (57)	14 (56)	8 (73)	8 (47)	
Overweight (25.0–29.9)	14 (26)	5 (20)	1 (9)	8 (47)	
Obese Class I (30.0–34.9)	8 (15)	5 (20)	2 (18)	1 (6)	

Abbreviations: py, pack years; ECOG, Eastern Cooperative Oncology Group; BMI, body mass index. ^1^ Analyzed by using Fisher’s exact test.

**Table 3:** Tumor characteristics of the study population (*n* = 53).

Tumor Characteristics	No. of Patients	Exon 19 Deletion	L858R Mutation	Uncommon Mutations	*p* Value ^1^
**Total**	53	25 (47%)	11 (21%)	17 (32%)	
**Stage at initial diagnosis**, *n* (%)					0.1449
Stage IIIB	4 (8)	1 (4)	-	3 (18)	
Stage IVA	22 (42)	8 (32)	5 (45)	9 (53)	
Stage IVB	27 (51)	16 (64)	6 (55)	5 (29)	
**Histologic type**, *n* (%)					0.341
Adenocarcinoma	48 (91)	24 (96)	10 (91)	14 (82)	
Squamous cell carcinoma	3 (6)	-	1 (9)	2 (12)	
NOS	2 (4)	1 (4)	-	1 (6)	
**PDL-1 status**, *n* (%)					0.5045
Negative (<1%)	25 (47)	11 (44)	8 (73)	6 (35)	
Low expression (1–49%)	21 (40)	11 (44)	3 (27)	7 (41)	
High expression (50–89%)	2 (4)	1 (4)	-	1 (6)	
Very high expression (≥90%)	1 (2)	1 (4)	-	-	
Unknown	4 (8)	1 (4)	-	3 (18)	
**Location of metastasis**, *n* (%)					0.8236
Pleura effusion	22 (42)	12 (48)	4 (36)	6 (35)	
Brain	14 (26)	9 (36)	2 (18)	3 (18)	
Bones	18 (34)	10 (40)	3 (27)	5 (29)	
Distant lymph nodes	5 (9)	3 (12)	-	2 (12)	
Adrenal gland	3 (6)	1 (4)	1 (9)	1 (6)	
Liver	6 (11)	4 (16)	1 (9)	1 (6)	
Other	5 (9)	1 (4)	3 (27)	1 (6)	
**Sites of metastasis**, *n* (%)					0.1079
No distant metastasis	4 (8)	1 (4)	-	3 (18)	
1	29 (55)	11 (44)	9 (82)	9 (53)	
2–3	20 (38)	13 (52)	2 (18)	5 (29)	

NOS, not otherwise specified; ^1^ analyzed by using Fisher’s exact test.

**Table 4:** Treatment pattern and response of the study population (*n* = 53).

Treatment Pattern and Response	No. of Patients	Common	Uncommon
**Total**	53	36 (68%)	17 (32%)
**TKI**, *n* (%)			
Afatinib (second-generation)	17 (32)	11 (31)	6 (35)
Brigatinib ^1^ (second-generation)	1 (2)	-	1 (6)
Osimertinib (third-generation)	31 (58)	25 (69)	6 (35)
Mobocertinib ^2^ (second-generation)	4 (8)	-	4 (24)
**Best response**, *n* (%)			
Complete response (CR)	-	-	-
Partial response (PR)	19 (36)	10 (28)	9 (53)
Stable disease (SD)	6 (11)	3 (8)	3 (18)
Progressive disease (PD)	2 (4)	2 (6)	-
Unknown ^3^	26 (49)	21 (58)	5 (29)

TKI, tyrosine kinase inhibitor; ^1^ TKI for ALK translocations; ^2^ TKI for exon 20 insertion; ^3^ “Unknown” indicates missing documentation of best tumor response and does not reflect missing survival or follow-up data.

**Table 5:** Median overall survival time in months according to EGFR subtypes (exon 19 deletion, exon 21 L858R mutation, uncommon mutations).

EGFR Subtype		95% Confidence Interval	
	Median	Lower Bound	Upper Bound
Exon 19 deletion	32.5	30.8	34.3
L858R mutation	17.4	17.0	18.1
Uncommon ^1^	11.6	9.2	14.0
Overall (*n* = 53)	17.7	10.4	25.0

^1^ Uncommon mutations: exon 18–21.

**Table 6:** Univariate and multivariable analysis for overall survival of NSCLC patients who received EGFR-TKI (first-line) and harbored an EGFR common or uncommon mutation (*n* = 53).

Variable	Overall Survival			
	Univariate		Multivariable	
	HR (95% CI)	*p* Value	HR (95% CI)	*p* Value
Age (<65 vs. ≥65)	1.0 (0.99–1.1)	0.099	2.50 (0.65–9.7)	0.185
Sex (male vs. female)	0.55 (0.26–1.2)	0.128	0.37 (0.13–1.1)	0.072
Histologic type (ADC vs. other)	4.3 (1.4–14)	0.013	1.59 (0.36–7.0)	0.542
BMI (<25 vs. ≥25)	0.77 (0.35–1.7)	0.517	0.63 (0.24–1.7)	0.355
ECOG status (0 vs. 1–2)	2.3 (1.1–4.5)	0.018	1.73 (0.74–4.0)	0.205
Sites of metastasis (≤1 vs. >1)	1.1 (0.52–2.4)	0.75	1.45 (0.54–3.8)	0.458
PDL-1 expression (0–49% vs. 50–100%)	0.8 (0.19–3.4)	0.765	0.78 (0.11–5.7)	0.808
EGFR mutation * (common vs. uncommon)	3.7 (1.6–8.7)	0.002	3.71 (1.23–11.2)	0.02

Abbreviations: HR, hazard ratio; EGFR, epidermal growth factor receptor; ECOG, Eastern Cooperative Oncology Group; BMI, body mass index; ADC, adenocarcinoma. * common mutations: EGFR exon 19 deletion, EGFR exon 21 L858R mutation; uncommon mutations: EGFR exon 20 insertion, EGFR exon 21 L861Q mutation, EGFR exon 20 S768i mutation.

**Table 7:** Median progression-free survival time in months according to EGFR subtype (exon 19 deletion, exon 21 L858R mutation, uncommon mutations).

EGFR Subtype		95% Confidence Interval	
	Median	Lower Bound	Upper Bound
Exon 19 deletion	23.1	6.8	39.4
L858R mutation	17.0	5.2	28.7
Uncommon ^1^	10.0	3.4	16.4
Overall	14.2	7.4	21.0

^1^ Uncommon mutations: exon 18–21.

**Table 8:** Univariate and multivariable analysis for progression-free survival of NSCLC patients who received EGFR-TKI (first-line) and harbored an EGFR common or uncommon mutation (*n* = 53).

Variable	Progression-Free Survival			
	Univariate		Multivariable	
	HR (95% CI)	*p* Value	HR (95% CI)	*p* Value
Age (<65 vs. ≥65)	2.2 (0.86–5.6)	0.1	2.59 (0.78–8.7)	0.122
Sex (male vs. female)	0.58 (0.26–1.3)	0.184	0.41 (0.15–1.1)	0.075
Histologic type (ADC vs. other)	2.5 (0.7–8.6)	0.16	1.21 (0.25–5.8)	0.808
BMI (<25 vs. ≥25)	1.1 (0.47–2.4)	0.883	1.48 (0.58–3.8)	0.417
ECOG status (0 vs. 1–2)	1.2 (0.56–2.5)	0.654	0.66 (0.27–1.7)	0.379
Sites of metastasis (≤1 vs. >1)	0.76 (0.32–1.8)	0.529	1.18 (0.43–3.2)	0.751
PDL-1 expression (0–49% vs. 50–100%)	0.38 (0.05–2.9)	0.35	0.24 (0.03–2.3)	0.218
EGFR mutation * (common vs. uncommon)	2.2 (0.97–5.2)	0.06	1.79 (0.60–5.3)	0.292

Abbreviations: HR, hazard ratio; EGFR, epidermal growth factor receptor; ECOG, Eastern Cooperative Oncology Group; BMI, body mass index; ADC, adenocarcinoma. * common mutations: EGFR exon 19 deletion, EGFR exon 21 L858R mutation; uncommon mutations: EGFR exon 20 insertion, EGFR exon 21 L861Q mutation, EGFR exon 20 S768i mutation.

## Data Availability

The data presented in this study are available on reasonable request from the corresponding author.
